# Advice about Work-Related Issues to Peers and Employers from Head and Neck Cancer Survivors

**DOI:** 10.1371/journal.pone.0152944

**Published:** 2016-04-12

**Authors:** Carolyn S. Dewa, Lucy Trojanowski, Sietske J. Tamminga, Jolie Ringash, Maurene McQuestion, Jeffrey S. Hoch

**Affiliations:** 1 Centre for Research on Employment and Workplace Health, Centre for Addiction and Mental Health, Toronto, Ontario, Canada; 2 Department of Psychiatry and Behavioral Sciences, University of California Davis, Sacramento, California, United States of America; 3 Institute of Health Policy, Management and Evaluation, University of Toronto, Toronto, Ontario, Canada; 4 Coronel Institute of Occupational Health Academic Medical Centre, University of Amsterdam, Amsterdam, The Netherlands; 5 Princess Margaret Cancer Centre, University Health Network, Toronto, Ontario, Canada; 6 Department of Public Health Sciences, University of California Davis, Davis, California, United States of America; Aichi Cancer Center Research Institute, JAPAN

## Abstract

**Purpose:**

The purpose of this exploratory and descriptive study is to contribute to the sparse return-to-work literature on head and neck cancer (HNC) survivors. Interview participants were asked to reflect upon their work-related experience with cancer by answering two specific questions: (1) What advice would you give someone who has been newly diagnosed with head and neck cancer? (2) What advice would you give to employers of these people?

**Methods:**

Data were gathered through 10 individual semi-structured in-depth interviews with HNC clinic patients at a regional cancer center’s head and neck clinic in Ontario, Canada. A constant comparative method of theme development was used. Codes identified in and derived from the data were discussed by research team members until consensus was reached. Codes with similar characteristics were grouped together and used to develop overarching themes.

**Results:**

Work-related advice for peers focused on personal self-care and interactions within workplaces. Work-related advice to employers focused on demonstrating basic human values as well as the importance of communication.

**Discussion:**

The study results suggest HNC clinic patients should be proactive with employers and help to set reasonable expectations and provide a realistic plan for work to be successfully completed. HNC clinic patients should develop communication skills to effectively disclose their cancer and treatment to employers.

**Conclusions:**

In this exploratory study, HNC clinic patients’ advice was solution-focused underscoring the importance of self-care and pro-active communication and planning with employers. Employers were advised to demonstrate core human values throughout all phases of the work disability episode beginning at diagnosis.

## Introduction

Since 1992, there has been a decline in Canada’s age-standardized total incidence rate for head and neck cancers from 16.8 per 100,000 in 1992 to 12.5 per 100,000 in 2009 [1]. In addition, approximately 61% of the people diagnosed were 60 years of age and older [1]. Furthermore, there is evidence suggesting that the probability of survival from the disease decreases with age [2]. Given that a large proportion of people who are diagnosed with head and neck cancer are retirement aged and present with lower survival rates, labor market participation has not been a prominent focus for this patient population. Together, these trends may help to explain why until recently, there has been a dearth of studies regarding the return-to-work experiences of head and neck cancer survivors. Indeed, in a systematic review of the literature, Mehnert [3] noted that the studies examining employment and work-related issues among cancer survivors predominantly focused on breast cancer survivors. Studies that have included head and neck cancer survivors suggest that this group may face more difficulty in returning to work than other groups of cancer survivors [4].

Indeed, while there was a decline in the total incidence rate for head and neck cancer, there has also been a significant increase in the incidence of specific types of head and neck cancers including human papillomavirus (HPV)-associated oropharyngeal squamous cell cancer [1, 5]. It has risen from 1.6 per 100,000 in 1992 to 2.6 per 100,000 in 2009 [1]. There is evidence suggesting that it could be rising by 4.9% per year [6]. In addition, the main groups affected have been men and those 50–59 years of age [1]. Furthermore, there has been a 13.5% increase in the survival rates for head and neck cancers [5], with an 82–89% survival rate for certain subgroups [7]. A recent study has found that over half of their sample population of head and neck survivors returned to work [8, 9]. Thus, the profile of head and neck cancer patients has been changing. They include more survivors and people who are working-aged. This supports a renewed interest in the return to work experiences of this population. These trends also explain the call for more research on return-to-work problems [10–13].

A number of systematic reviews indicate there exists a body of literature examining factors related to cancer survivor return-to-work (e.g., [4, 14, 15]). Few of these studies have included head and neck cancer survivors. In addition, most of the existing literature asks survivors about their experiences (e.g., [9]); few have asked survivors about their advice. There is a distinction between experience and advice. The dictionary defines advice as, “opinion or suggestion about what someone should do”. It asks respondents to reflect on actionable aspects of circumstances over which they perceive they have control. For this paper, we focus on two questions that interview participants were asked about their advice as it related to work. The specific questions were: (1) What advice would you give someone who has been newly diagnosed with head and neck cancer? (2) What advice would you give to employers of these people? That is, these questions ask respondents to identify specific aspects of their working that they could do something about as well as what they perceive their employers could do to make the experience easier. Because the focus is on actions, it is distinguished from asking about barriers and obstacles. Few published studies have taken this approach with this group of cancer survivors. Yet, there is evidence that survivors find the perceived support from the workplace to be important [9].

The purpose of this exploratory and descriptive study is to contribute to the sparse return-to-work literature on head and neck cancer survivors. Our approach seeks to fill a current gap in the occupational rehabilitation literature by broadening the understanding of the return-to-work process beyond the physical and functional effects of treatment. The type of information gathered in this study can contribute to the development of meaningful rehabilitation processes for head and neck cancer survivors. Data were gathered through individual in-depth interviews with patients at a head and neck cancer clinic in a regional cancer centre in Ontario, Canada’s most populous province.

## Methods

An exploratory and descriptive study using qualitative and quantitative methods was conducted. This study aims to obtain in-depth information on cancer survivors’ experiences of returning to work. In this study, we employ the definition of survivor used by the Cancer Centre. This includes people who are in post treatment, cured or at a point of transitioning care to another provider. It also extends to people who have been diagnosed with cancer who are still living with their cancer and need support services (i.e., those with a recurrence).

This research was reviewed and approved by the research ethics boards at the Centre for Addiction and Mental Health and Princess Margaret Cancer Centre. All interview participants provided written informed consent to participate and to be audio-recorded. We used the items of the Consolidated Criteria for Reporting Qualitative studies (COREQ) to improve the reporting of this qualitative study [16].

### The Context

In Canada, workers who have been employed at their workplaces for at least three weeks are protected for sickness related absences that do not exceed 17 weeks [17]. Workers who require a work absence that extends beyond what would be covered by “sick leave” must file an insurance claim for income replacement benefits which are often called disability benefits (i.e., short-term or long-term disability). These benefits may be either publicly or privately sponsored. In Canada, the federal government offers incentives to employers to provide disability benefits to workers [18]. These employer-sponsored work disability benefits not only provide income support during the absence, they also guarantee a position is waiting for the worker when s/he returns to work.

### Participant Recruitment

Recruitment for the study was conducted in the waiting room of a head and neck cancer clinic at a regional cancer centre in Ontario. Consecutive patients waiting for follow-up appointments in this clinic were approached by trained interviewers who explained the study and eligibility criteria. Eligibility criteria included: (1) currently working (i.e. either self-employed or by an employer), (2) diagnosed with head or neck cancer within the past five years, (3) working at time of diagnosis, and (4) fluency in English. The study consent form was reviewed with interested participants. After providing informed consent, participants were given a paper questionnaire to complete for the quantitative part of this study (to be reported separately). At that time, interviewers also informed participants about the qualitative component of the study. All participants who filled out the quantitative questionnaire were invited to provide their contact information if they were interested in taking part in the qualitative interview. However, they were not required to consent to being contacted for the qualitative interview to participate in the quantitative questionnaire.

A research assistant contacted interested participants through their preferred means of contact, either by telephone or email, to schedule an interview. Participants were offered a $30 honorarium for interview completion. There were 33 people out of the 80 patients who completed the quantitative survey who expressed interest in participating in a qualitative interview and provided their contact information. Recruitment took place from July 2014 to March 2015. Interviews continued until saturation was achieved. Saturation was determined when no new information was being drawn from the sample; this was confirmed by two members of the research team (CSD and LT). Guest et al. [19] suggest that saturation in qualitative interviewing can be achieved with a sample size of six to 12 participants. Saturation was reached with a total of 10 patients (five men and five women).

Interviews were conducted in November and December 2014, and February and March 2015 by the first author and lasted an average of approximately 60 minutes (range 33–89 minutes). The first author is a female professor, who is a trained interviewer and specializes in work and health research. The interviewer had no direct relationship with the participants nor did she represent an organization or health care profession in the field of cancer care or occupational care. Interviewees were interviewed only once face-to-face, without establishing a relationship with the interviewer before the interview. Interviews took place at a different institution from where participants were receiving treatment. Each participant was informed about the aim of the study, the fact that information would be handled in a confidential manner, and the position of the interviewer.

### Semi-Structured Interviews

The research question was informed by a review of the literature (e.g., [20–22]) and consultations with a knowledgeable research expert in the field (ST). The general focus of the interviews was participants’ work experiences before and after cancer diagnosis, and their perceptions of the scales included in the quantitative survey. A semi-structured interview guide was developed for use during the interviews to ensure that subject areas and topics explored during data collection would be consistent [23]. The semi-structured interview guide was not pilot-tested. However, throughout the course of data collection, probes related to emergent themes were added to the interview guide.

In the semi-structured interviews, participants were asked to consider five primary questions: (1) Can you describe your job? (2) How did you tell your employer about your diagnosis? (3) How did you decide to return to work? (4) What work-related advice would you give someone who has been newly diagnosed with head and neck cancer? (5) What advice would you give to employers? These analyses focused on the responses to the final two questions.

In the semi-structured interviews, in addition to the interview guide questions, additional questions emerged from the dialogue between the interviewer and study participant [24]. That is, the interview guide’s five questions did not change throughout the course of the data collection. Rather, because interviews were semi-structured and not limited to a structured interview format and guide, probe questions related to previous respondents’ answers were used to stimulate further conversation. As is a conventional method, in our semi-structured interviews, we concurrently engaged in the data collection and analysis processes, paying attention to similarities and repetition of themes [24]. Although there was some variation in the conversations between individual participants and interviewer, ultimately, the themes in the data were still common among interviews. The concurrent data collection and analysis allowed for the determination that a point of saturation had been achieved; this was determined when the newly collected data no longer revealed new insights or properties (i.e., saturation was achieved) [25].

### Analysis

The in-depth interviews were audio-recorded and transcribed verbatim. Field notes were made during and after the interviews. Transcripts were not returned to the participants for comments or corrections nor did participants provide feedback on our findings. As noted previously, a constant comparative method of theme development was used in which newly collected data were compared to already collected and coded data [26]. To establish the study’s internal validity, a triangulation approach was used such that three members of the research team (CSD, ST and LT) read all the transcripts and independently assigned codes to sections of text based on identified concepts. Once each individually completed this process, the coders came together to intensively discuss the codes assigned and to negotiate agreement and disagreement [27]. Because this collaborative coding approach was used, intercoder agreement was not calculated [27]. Following the example described by Harry et al. [28], the goal was to achieve consensus; thus, a numerical reliability rating was not used. Rather, each point of difference was debated and clarified until the group agreed on the set of codes. When there was a consensus of all three coders as to whether to include a code and how it should be framed, the code was included in further analysis. Codes with similar characteristics were grouped together into categories. In turn, categories led to the development of overarching themes [27].

## Results

The results are divided into two parts. The first part contains the description of the participant characteristics. The second part contains the themes that emerged from the qualitative interviews and are the product of the coding process.

### Description of participants

The description of participants is taken from the demographic section of the quantitative questionnaire filled out by all study participants. A total of 10 patients (five men and five women) participated in the qualitative interviews. The mean age of participants was 48 years (range: 23–66 years) at the time of the interview (**Table 1**). Half of the participants had annual incomes of ≥ $100,000. In one case, a family member of a participant was present during the interview.

**Table 1:** Participants’ Disease-related Characteristics.

			Treatment				
Participant	Age at Interview	Primary Cancer Diagnosis	Surgery	Radiation	Chemotherapy	Time Since Diagnosis	Current Status
100004	61	Salivary glands	X	X		819 days (26.9 months)	Completed treatment >6 months ago
100008	50	Hypopharynx	X	X		209 days (6.9 months)	Completed treatment <6 months ago, cancer has returned
100019	23	Oropharynx	X			735 days (24.1 months)	Completed treatment >6 months ago
100022	66	Oropharynx		X	X	568 days (18.7 months)	Completed treatment >6 months ago
100028	52	Other (kidney)	X			1146 days (37.7 months)	Completed treatment >6 months ago
100037	56	Oral cavity	X			1339 days (44.0 months)	Completed treatment >6 months ago
100043	29	Salivary glands	X	X		468 days (15.4 months)	Completed treatment >6 months ago
100046	65	Oropharynx		X	X	1058 days (34.8 months)	Completed treatment >6 months ago
100072	23	Thyroid	X	X	X	50 days (1.6 months)	In treatment
100078	59	Oral cavity		X	X	1018 days (33.4 months)	Completed treatment >6 months ago

#### Disease-related characteristics

The mean number of months between diagnosis and the interview was 24 months (**Table 1**). Three of the participants had a primary diagnosis of oropharyngeal cancer, two had cancer of the salivary glands, two had cancer of the oral cavity, one had thyroid cancer, one had hypopharyngeal cancer and one had kidney cancer. About 70% of the participants underwent surgery, 70% had radiation therapy and 40% received chemotherapy. One participant was receiving cancer treatment at the time of the interview.

#### Work-related characteristics

The participants were employed in a diversity of occupations in management, administration, sales and music/arts (**Table 2**). All but two of the participants were currently working full-time (ie., ≥ 32 hours/week). Four of the participants chose not to take disability leave because one had sufficient sick days so they could avoid taking a disability leave, one quit their job, one took vacation and one continued to work. For those who did take disability leave, the mean number of disability leave days was 181 days with a range of 57–305 days.

**Table 2:** Participants’ Work-related Characteristics.

	Work Status at Time of Interview		Return-to-Work	
Participant	Occupation	Work Hours (Per Week)	Disability Leave During Treatment	Currently at Pre-diagnosis Job
100004	Administrative, financial or clerical	40	223 days (7.3 months)	Yes
100008	Management	50	98 days (3.2 months)	Yes
100019	Sales or service	20	150 days (4.9 months)	No
100022	Trades, transport, construction or equipment operator	42	250 days (8.2 months)	Yes
100028	Administrative, financial or clerical	35	57 days (1.9 months)	Yes
100037	Professional	45	None–Sick leave (0.5 months)	Yes
100043	Music/arts	4	None–Vacation	Yes
100046	Management	60	305 days (10.0 months)	Yes
100072	Music/arts	50	NA–Left job	No
100078	Management	45	None–Continued to work	Yes

### Themes from the Qualitative Interviews: Advice to Peers

The responses to the first study question about advice to peers clustered into two sets of themes. The first set focused on the individual. These themes included: (1) take responsibility for your own health and future and (2) prioritize your health. The second set clustered around interactions between the individual and their workplaces. These themes included: (1) be proactive regarding your work and (2) seek support.

#### Take responsibility for your own health and future

There were a number of ways that participants identified how someone could take responsibility for their own health. One was to protect one’s own health. For example, one respondent urged, “…for your own body and for yourself make sure what you’re doing is going to contribute to better health and get better.” P100022. Another respondent spoke about the importance of creating one’s own safety net such as getting disability insurance. He advised, “I’d certainly advise them to have disability insurance. I would advise them to try to find a policy that will cover them in all situations.” P100046. But, he also cautioned that there are no guarantees despite the fact that someone plans ahead because it is difficult to plan for every circumstance.

#### Prioritize your health

A related theme that emerged was prioritizing one’s own health over returning to work. There was recognition that returning to work and returning to health are not synonymous. The respondents emphasized that people might be able to return to work before they are completely healthy. One respondent related, “You really have to allow yourself the opportunity to properly heal. Don’t push going back to work too soon. I put a lot of expectation on myself. So, I wanted to … I was pushing myself because I wanted to get back to normal.” P100008. Although there might be a strong desire to return to pre-diagnosis states, acting upon the desire before a person is ready can be harmful in the long-run.

Oncologists were also viewed as important sources of information regarding one’s health status. Thus, they should be a part of the decision regarding whether someone is ready to return to work. There was also the acknowledgment that many well-meaning people would offer advice. Yet, it is important not to succumb to peer pressure. As a respondent rationalized, “You may receive a lot of opinions about what you will be going through, but let your doctor be the judge of everything, because he is the one who is going to see you when you go for your treatment, and he will be the best judge.” P100004. Oncologists may contribute by helping people set realistic expectations about how they will be affected by treatment side-effects and recovery time.

#### Be proactive about work

Respondents suggested that it is essential to reassure the employer that the employee understands the employer’s perspective. To that end, respondents suggested that people offer their employers a realistic plan before the illness and treatment affects work. They also emphasized the importance of communication. One respondent warned, “What shouldn’t you do? Well, I think an employee should be up front and realistic with what they can do and what they are not able to do. So, if, as an employee, you just obfuscate and you are not providing reasonable information, it becomes, I think, difficult for an employer to know where they stand.” P100037. As part of providing assurance, respondents also pointed out the need to confirm that returning to work is part of their plans.

The respondents also suggested that there is a balance to be struck. This means being aware of what one wants when communicating with an employer. The employee should not depend on the employer to control the discussion. This involves thinking through what s/he is comfortable sharing with the employer. One respondent described, “Well, I think it’s really a question of how much you need to share in order to, at the end of the day, ultimately satisfy what you want. Some people would treasure their personal privacy more than the ability to do the job in a way that they would like to do. So some people will balance self-disclosure over maximum flexibility in doing their job or even in the case of having a job.” P100028. There is an assumption that with more information, there is more understanding. In turn, this will lead to more flexibility if the employee is experiencing difficulty at work. At the same time, there was also the acknowledgment that this assumption may not hold true for all employers.

#### Seek support

Most of the participants expressed a fierce sense of pride. They did not want to be pitied. They did not want to appear weak. At the same time, there was the recognition that there were times when they needed help. Their advice was, “Lower your pride. Allow people to help you.” P100008. Help could take the form of either physical help or emotional support.

### Themes from the Qualitative Interviews: Advice to Employers

The advice to employers fell into two primary themes. The first focused on the practical things that employers could do to make the disability benefit application process more compassionate. Suggestions clustered around: (1) reporting burden and (2) timing.

The second themes were focused on how employers should interact with employees who have been diagnosed with cancer and are receiving treatment. These included: (1) privacy, (2) respect (3) flexibility and (4) honesty.

#### Adopt a compassionate disability benefit application process

Difficulties with the disability benefit application were among the concerns that respondents voiced. They wanted employers to be aware of the need for a compassionate process. Respondents faced the greatest challenge when they were required to apply for benefits when they were in treatment. One respondent described, “…one thing that I found is making the process of getting the benefits easier on that person who is going through a medical condition because one of the things that I found stressful was, here I was, going through radiation treatment, and I was running around all over the city getting forms signed and faxed.” P100008. The solutions included making the process streamlined, offering support to gather the requisite medical information, thus allowing for adequate time to complete the application.

In addition, when the company is collecting information, it should be more aware of where they are calling. A respondent gave the example of the company calling the hospital to speak to the employee just after surgery. She wondered whether the call could have waited until she was discharged and at home.

#### Be aware of privacy

Privacy was another concern that respondents discussed. The advice for employers was, “Maybe not ask a whole lot of questions about the diagnosis. It might be difficult for the person to talk about. It might be very personal. Instead, they [the employer] could say, I'm here if you want to talk about it, or something along those lines…There, obviously, needs to be a conversation, but also respect their privacy a little bit.” P100043. Respondents believed that it was important for employers to allow the employee to decide the amount of detail to provide.

#### Be respectful

The underlying concept with regard to respect was the willingness to recognize the individual needs of the employee. This was described with, “Well, I appreciated, with my employer, particularly, that … they reacted neutrally … some people want positive reinforcement or support, in that sense. But I’m glad that they were neutral about it because that’s what I appreciate. It didn’t show that they were emotional about it, that they just dealt with it, like, okay, this is that and we’ll deal with it accordingly.” P100072. This suggests that there is variation in the levels and types of support that employees want.

One of the things that the respondents felt strongly against was pity. People did not want pity. They wanted to continue to contribute to their organizations. Thus, they did not want employers to automatically assume that “the person is not going to be able to work at all, or is going to be undependable all of a sudden, or, that they’re suddenly a different person, everything has changed all of a sudden.” P100043. This led respondents to suggest the importance of work accommodations and communication. Acknowledging that accommodations can be difficult, respondents wanted employers to try to find the balance of being empathetic and practical. An effective process was described as, “I think if you have a supportive management team who will look at the individual, look at what needs to be done, look at the job and accommodate accordingly, that really makes a big difference.” P1000037. At the core is recognizing the worker as an individual and starting with the premise that s/he can contribute.

#### Be flexible

Work accommodations also involve flexibility. Respondents described flexibility as a willingness to help. They described it as looking at what needs to be done and identifying what the employee can successfully accomplish. They suggested that another goal of the flexibility is to ensure that the employee feels there is a meaningful role. It also translates into employees being able to ask for help and expect that they can get help.

#### Be honest

Finally, respondents felt that it was important for employers to be clear about how they could help employees as well as being clear about their constraints and their expectations for the employee. One respondent described,

*I think if you lay down the line… what you can do for your employee*, *what you can’t do for an employee then you have a framing of the situation that would give the employee the latitude to decide what he or she wants to disclose*, *what he or she needs in terms of accommodation*. *If you don’t have something in the personnel manual that lays it out*, *if HR doesn’t have some kind of definitive statement*, *then I think it will just create a more psychological stress for both parties because one doesn’t want to ask too much and the other doesn’t want to hear too much… So you’re not creating a wholesome workplace environment if you don’t spell out things*. *P1000028*

The point that respondents seemed to be making was that it is important to communicate rather than to assume. They wanted employers to be open to talk about how they could continue to contribute. They wanted to be trusted to relay the necessary information. They thought that employers should also be honest about their expectations.

## Discussion

In this study, we asked head and neck cancer survivors to reflect on their work-related experiences with cancer and to offer advice to: (1) workers who have been newly diagnosed with head and neck cancer and (2) employers of those workers. Advice that would be offered to peers focused on two aspects. One involved personal self-care responsibilities; the other involved interactions with workplaces and others. With regard to self-care responsibilities, respondents reinforced the importance of self-care and self-awareness. That is, they recommended that people make their own health a priority and take the responsibility for their own care.

However, this did not mean that they should do this alone. On the contrary, they recommended that their oncologists should be considered the main source of advice and information; they felt this was important given the potential for other numerous sources of advice. This underscores the importance of the oncologist’s role in the return-to-work process. Oncologists and other members of the patient’s healthcare team (i.e., nurses, social workers, speech language pathologists and occupational therapists) could be important sources of information for newly diagnosed workers about how their work ability will be affected and what employers should be told. It also suggests a need for physicians to be trained about how to advise workers especially at diagnosis. However, given that there is equivocal evidence regarding the impact of physician advice on the return-to-work process [29, 30], this also suggests there are opportunities for more research about identifying the most effective content of the advice.

The respondents also seemed to differentiate between peer advice and support. Although they seemed to hesitate to accept advice, they encouraged accepting support from others. With this, they acknowledge that receiving support can be difficult because of the temporary vulnerability that necessitates help both physical and psychological. At the same time, it should be noted that employers will often prioritize practical and concrete measures over emotional support [31]. Thus, the employer may not be an effective source of emotional support.

Perhaps, one of the ways to address their vulnerability is by being proactive with the workplace. Respondents described this as entailing helping the employer to set reasonable expectations by being honest and by providing a realistic plan about how to get the work successfully completed. This advice is similar to the best practice guidelines for employers who have workers returning to work from a mental disorder-related disability leave [32]. This advice also addresses some of the concerns that have been expressed by employers regarding their desire for more information about the uncertainty surrounding a cancer diagnosis; they want information about prognoses and anticipated work absences [33]. Employers have suggested that this type of communication helps to build mutual bonds and the feeling that the return-to-work is a collaborative effort [33]. It also avoids leaving the process to trial and error [31]. This also suggests that there is a need to help cancer survivors to develop the skills about how to disclose their cancer and treatment to give employers sufficient information to be able to respond to their needs [21, 22]. This type of training may be particularly useful in situations where survivors have difficulty in explaining the work-related impact of their treatment [31]. Indeed, there is evidence that some survivors find it difficult to explain their limitations and to ask for help [22].

In contrast to what we expected based on the literature that found oral dysfunction and anxiety were associated with RTW [8], the advice that the participants offered to peers does not seem to be specific to the head and neck cancer survivors. Although, participants did mention specific head and neck cancer related side effects (e.g. speech problems) as barrier for their return to work, they did not include this in their advice to peers. This could be explained by the fact that rather than focus on the limitations they encounter arising from treatment (e.g. appetite, difficulty swallowing, speech impediments) [8], they focused on solutions (e.g. take responsibility for your own health). That is, when the questions about the types of advice they would offer were posed, they sought to offer solutions to the side-effects that accompany treatment. This also means that they did not focus on the things that they did not believe could be resolved.

One of the novel aspects of this study is its exploration of the advice survivors would offer to employers. It is one of the first studies that have offered head and neck cancer survivors an opportunity to voice their recommendations to employers. These findings may begin to answer another major concern of employers; they do not always know what to ask, how to help, and how to show concern [33].

The advice that the study participants offered to employers does not seem to be specific to the head and neck cancer diagnosis. Rather, they are principles that reflect basic human values such as compassion, empathy, honesty, and respect. The message that participants seem to convey is that employers should communicate with their employees. This approach could help employers more effectively manage their own conflicting roles of being a representative of the organization’s best interest and being responsible for their employee [33]. At the same time, in the literature, employees have expressed concern about where the line between support and invasion of privacy should be drawn [31]. Honest and respectful communication can help employers and employees find where the boundaries lie because the boundaries can be different for each employee. This type of communication can also help both parties understand expectations and encourage return-to-work [34].

Interestingly, the advice from these cancer survivors to employers reflects the best practice guidelines for employers who have workers returning to work from a mental disorder-related disability leave [32]. There is an emphasis on work accommodation that is flexible, supportive, and developed collaboratively with the worker and employer. In fact, it seems that employers understand the need for flexibility and graduated return-to-work [34]. It also has been suggested that managers should be provided with training on strategies to help workers set realistic expectations of themselves and how to understand the help needed [31]. From this study, there also seems to be a need for flexibility and accommodation at all phases of the work disability episode starting at diagnosis and the disability application.

### Limitations

These results should also be considered with respect to the limitations of the study. First, because this is an exploratory and descriptive study, the results may not be generalizable to all head and neck cancer survivors. Future research should consider exploring the experiences of head and neck cancer patients who are receiving treatment in other clinics to compare and contrast the results reported in this study. For example, the participants in this study were highly educated and were employed in work situations that allowed them to participate in the interviews. This is a limitation associated with volunteer bias to which most research is subject. Thus, people who did not think they were able to take time away from work would not have been included in the study. It will be important for future studies to include workers in more constrained employment situations.

Because the recruitment was conducted in the clinic waiting room, people may have felt compelled to participate to express their appreciation for the clinic care they received. In an effort to address this possibility, the research assistant who called interested participants to schedule the interviews, identified her affiliation as being with another institution. In addition, during this phone call, she clarified that their cancer clinical care provider would not be aware of their participation in the study. At that time, they should have felt free to decline to participate in the qualitative interview.

Fluency in spoken English was one of the requirements for participation in the study. This inclusion criterion may have disadvantaged new immigrants and non-English speaking people from participating in the interviews. At the same time, we were able to recruit a culturally diverse set of participants who could reflect different cultural influences. However, future studies should include opportunities for the participation of new immigrants and non-English speaking people; their experiences with navigating both new employment and healthcare systems may be different from those of this present sample.

Finally, work disability is complex. As Loisel et al. [35] point out, it involves a least four distinct systems: (1) healthcare, (2) legislative and insurance, (3) personal, and (4) workplace. In this regard, we asked respondents to focus on two systems–the personal and workplace. There are other systems and mechanisms through these systems that can be used to improve the work disability experience. Future research could include these systems.

## Conclusions

In this exploratory study, head and neck cancer survivors were asked about the advice they would give to peers who have recently been diagnosed with head and neck cancer and to their employers. Their responses were solution-focused. Their advice to their peers underscored the importance of self-care, the oncologist’s advice and pro-active communication and planning with employers. This advice also suggested areas for future inquiry that include the types of work-related advice for clinicians to offer and communications skill training for workers about what and how they should tell their employers about their diagnoses, treatment and their effects on their work abilities.

Their advice to employers emphasized core human values such as compassion, empathy, honesty, and respect. They suggested these could be demonstrated in all phases of the work disability episode starting at diagnosis and the disability application and continuing on to return-to-work. Their advice also suggested the role for skills training to help managers be effective in helping workers to remain productive.

## Compliance with Ethical Standards

### Research involving Human Participants

All procedures performed in studies involving human participants were in accordance with the ethical standards of the institutional research committee and with the 1964 Helsinki declaration and its later amendments or comparable ethical standards.

### Informed consent

Informed consent was obtained from all individual participants included in the study.
